# Methotrexate therapy as a promising long-term treatment approach for immune-mediated adverse reactions of tofersen in *SOD1*-ALS: a case report

**DOI:** 10.1007/s00415-025-13415-3

**Published:** 2025-10-27

**Authors:** Maximilian Vidovic, Hanna Sophie Lapp, Isabelle Dittes, Nicolai Leuchten, Martin Aringer, Claudia Günther, René Günther

**Affiliations:** 1 Department of Neurology, Faculty of Medicine and University Hospital Carl Gustav Carus, TUD Dresden University of Technology, Fetscherstraße 74, 01307 Dresden, Germany; 2 Department of Rheumatology, Faculty of Medicine and University Hospital Carl Gustav Carus, TUD Dresden University of Technology, Fetscherstraße 74, 01307 Dresden, Germany; 3 University Center for Autoimmune and Rheumatic Entities (UCARE), Faculty of Medicine and University Hospital Carl Gustav Carus, TUD Dresden University of Technology, Fetscherstraße 74, 01307 Dresden, Germany; 4 Department of Dermatology, Faculty of Medicine and University Hospital Carl Gustav Carus, TUD Dresden University of Technology, Fetscherstraße 74, 01307 Dresden, Germany; 5 German Center for Neurodegenerative Diseases (DZNE), Tatzberg 41, 01307 Dresden, Germany

**Keywords:** *SOD1*-ALS, Tofersen, Adverse reactions, Autoimmune, Immune-mediated, Myelitis, Immunosuppression, Methotrexate

Dear Sirs,

Amyotrophic lateral sclerosis (ALS) is a neurodegenerative disease characterized by progressive loss of motor neurons, leading to weakness of the voluntary muscle system and ultimately to death due to respiratory failure. Ten-to-fifteen percent of the patients have a family history of ALS, known as familial ALS (fALS), whereas approximately 85% of the cases occur sporadically, defined as sporadic ALS (sALS) [1]. Disease-causing mutations in the *superoxide dismutase 1* (*SOD1*) gene are found in approximately 12% of fALS and 2% of sALS cases in Europe [2]. A recent study indicates that pathogenic and likely pathogenic *SOD1* variants represent about 2% of all ALS cases [3]. With tofersen, the first antisense oligonucleotide (ASO) therapy for ALS was introduced [4, 5]. It was granted for accelerated approval by the U.S. Food and Drug Administration (FDA) in April 2023. In Germany and other European countries, it was offered via an early access program (EAP) from January 2022 to June 2024 and was authorized by the European Medicines Agency (EMA) in May 2024. Tofersen treatment consistently demonstrated a significant reduction in neurofilament light chain (NfL) levels [5–8]. Recent “real-world” data confirmed a relevant decline of disease progression, clinical stabilization, and even some improvement of motor function [8, 9]. Clinically asymptomatic alterations in cerebrospinal fluid (CSF) were frequently observed in patients receiving tofersen. These included pleocytosis, elevated protein levels, intrathecal immunoglobulin synthesis, and CSF-specific oligoclonal immunoglobulin G (IgG) bands. Such findings suggest a potential autoinflammatory response to the ASO treatment [6, 10, 11]. Cytological analyses of the CSF revealed phagocytic cells with unidentified inclusion bodies. Some of these were located near to lymphocytes, potentially indicating an immune reaction [12]. Although serious adverse events related to tofersen are rare, complications, such as aseptic meningitis, myelitis, polyradiculitis, increased intracranial pressure, and papilledema, have been reported [5, 6, 13]. In a case of myelitis during tofersen treatment, rapid reduction of elevated intrathecal markers and clinical improvement were observed after prednisolone therapy, underlining the hypothesis of an autoinflammatory cascade. However, tofersen treatment was not resumed, leaving the mid- and long-term outcomes uncertain [13]. Yet, these immune-mediated clinical and laboratory findings have not been systematically investigated, and no standardized treatment approach for managing these adverse events has been established. Here, we present a case of a patient with *SOD1*-ALS who developed an immune-mediated adverse reaction during tofersen treatment, emphasizing the importance of considering this adverse event and illustrating the benefits and effects of immunomodulatory therapies.

## Case presentation

We investigated a 40 year-old female patient with ALS and a confirmed likely pathogenic *SOD1* variant (c-358-10T > G, heterozygous). Her family history was negative for the disease. The first clinical signs occurred in July 2021 with muscle weakness of the upper right extremity. The patient was diagnosed with ALS in March 2022, followed by genetic diagnosis in August 2022. By the time of diagnosis, the ALS functional rating scale in its revised form (ALSFRS-R) was 37 out of 48 points. Later, she developed paraparesis of the lower extremities. At this stage, there was no clinical evidence of bulbar motor signs. The patient was included in the tofersen EAP in October 2022 and subsequently transitioned to regular treatment following its approval. The EAP included a dosing phase with intrathecal administration of 100 mg tofersen at days 1, 14, and 28, followed by a maintenance phase including 29 doses at a median interval of 29 days (range: 25–38 days). The most recent dose was administered in March 2025.

### Immune-mediated adverse events

**Fig. 1:**
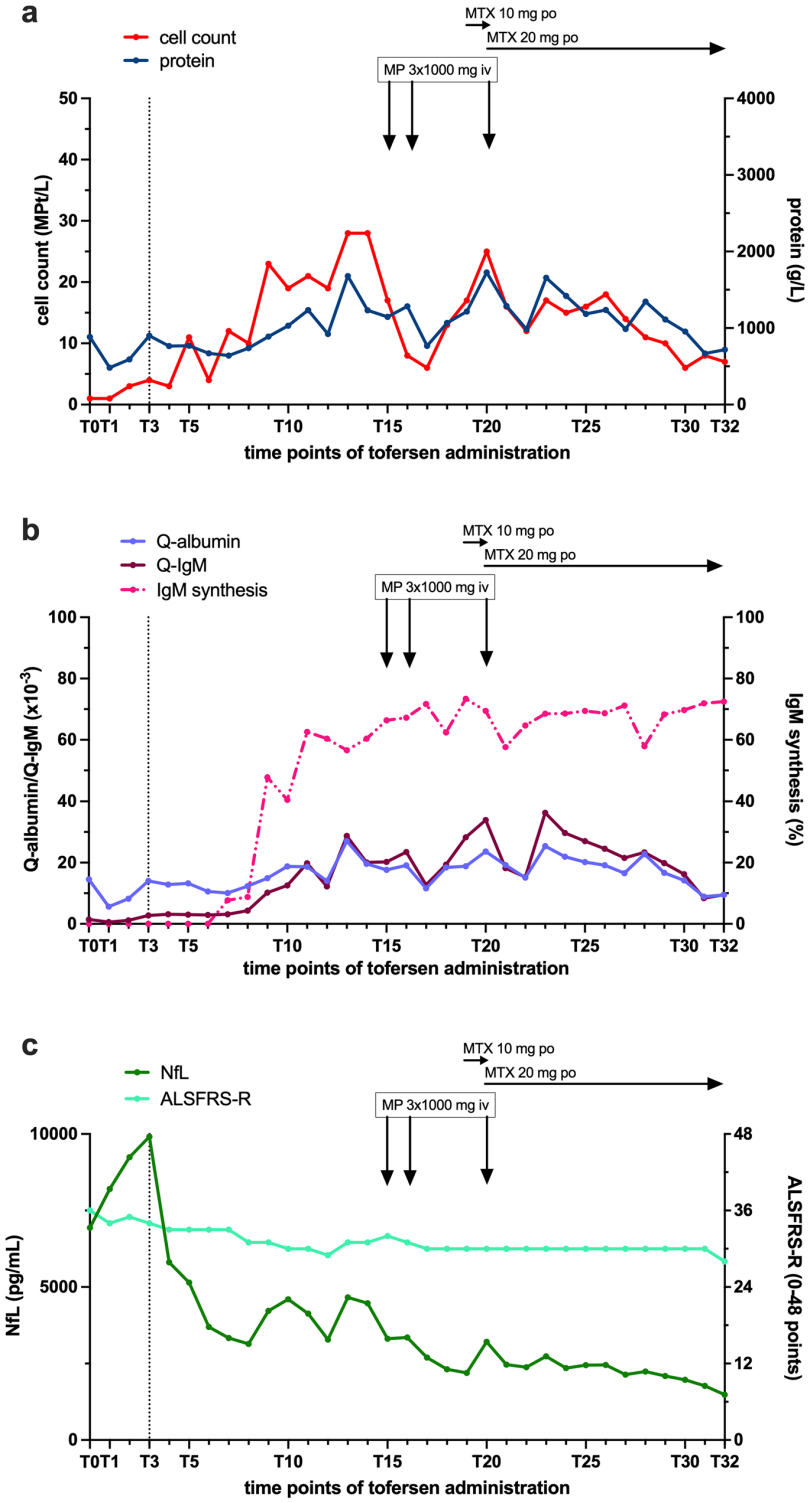
Laboratory findings of a patient with *SOD1*-ALS during treatment with tofersen. T0, pre-treatment; T1 to T3, dosing phase with intrathecal administration of 100 mg tofersen at day 1 (T1), 14 (T2) and 28 (T3); T4 to T32, maintenance phase with intrathecal administration of 100 mg tofersen including 29 doses at a median interval of 29 days (range: 25–38 days). Black dotted vertical line indicates the start of the maintenance phase. Intravenous administration of 1000 mg MP for 3 consecutive days at T15, T16, and T20. Oral administration of MTX 10 mg per week from T19 and 20 mg per week from T20. **a** Cell count (red) and protein concentration (blue) in CSF. **b** Q-IgM (dark red), Q-Albumin (lilac) and intrathecal IgM synthesis (dashed pink). **c** NfL concentration in CSF (dark green) and ALSFRS-R (light green)**.** *ALSFRS-R* ALS functional rating scale in its revised form, *CSF* Cerebrospinal fluid, *NfL* Neurofilament light chain, *MP* Methylprednisolone, *MTX* Methotrexate, *IgM* Immunoglobulin M, *iv* Intravenous administration, *po* Oral administration, *Q-albumin* CSF/serum ratio of albumin, *Q-IgM* CSF/serum ratio of IgM, *SOD1*-*ALS* *superoxide dismutase 1*-linked amyotrophic lateral sclerosis

**Fig. 2:**
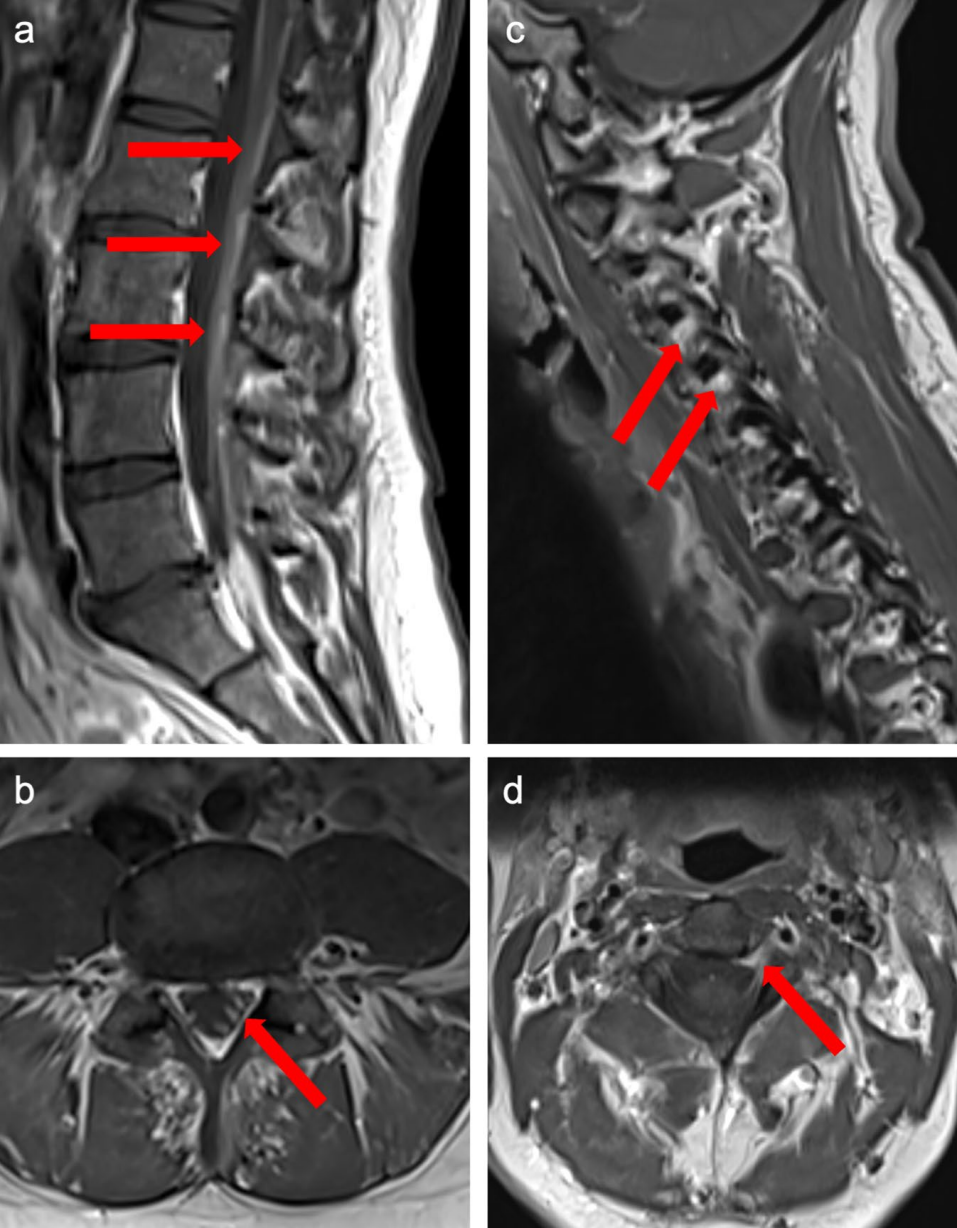
MRI findings of a patient with *SOD1*-ALS during treatment with tofersen. Red arrows indicate contrast enhancement. **a** Sagittal view of the lumbar spine. **b** Transversal view of the lumbar spine. **c** Sagittal view of the cervical spine. **d** Transversal view of the cervical spine. *SOD1*-*ALS* *superoxide dismutase 1*-linked amyotrophic lateral sclerosis

The patient suffered from progressively worsening, prolonged holocephalic headache after the third tofersen administration (T3). Between T20 and T23, she experienced transient episodes of fever, shivering, fatigue, and radiating pain in the lower extremities after each intrathecal administration. A gradually increasing pleocytosis and elevating protein levels were evident after initiation of tofersen administration (Fig. 1a, b). Microbiological and virological studies excluded an underlying infection of the central nervous system. Additionally, intrathecal immunoglobulin M (IgM) synthesis was first observed, and the CSF/serum IgM ratio (Q-IgM) continued to increase after T7. A spinal MRI scan conducted just before T15 revealed contrast enhancement in the caudate fibers (Fig. 2a, b) and partial involvement of the cervical roots (Fig. 2c, d).

### Initiation of concomitant methylprednisolone treatment

Due to the laboratory and radiological findings, and the symptoms suggestive of an immune-mediated reaction, the patient received 1000 mg intravenous methylprednisolone (MP) daily for three consecutive days at T15 and T16. Subsequently, the cell count decreased from 17 to 6 MPt/L and intrathecal IgM synthesis decreased from 72 to 62% by T18. After T17, the cell count re-increased from 6 to 17 MPt/L by T19. Similarly, Q-IgM rose from 12 to 34, and intrathecal IgM synthesis increased from 62 to 73%.

### Initiation of concomitant methotrexate treatment

In response, immunosuppressive oral therapy with methotrexate (MTX) was initiated at a dose of 10 mg per week starting at T19. Due to rising cell counts, lack of clinical improvement, and a renewed increase in NfL, the dose was raised to 20 mg per week at T20. Furthermore, a third administration of 1000 mg MP over 3 days was provided at T20. In response, the cell count decreased from 25 to 16 MPt/L. Q-IgM decreased from 34 to 18, while intrathecal IgM synthesis declined from 69 to 57% at T21. After T21, pleocytosis initially remained stable before gradually decreasing, reaching the lowest cell count of 6 MPt/L at T30. Q-IgM also gradually decreased from 36 at T23 to 9 at T32. Levels of the intrathecal IgM synthesis fluctuated between approximately 57% and 71% since the initiation of MTX medication at T19. No relevant increase has been observed after T22. All symptoms gradually resolved and responded to concomitant non-steroidal anti-inflammatory treatment without relapse, suggesting that they were clinical manifestations of aseptic meningitis and polyradiculitis with neuralgia. MTX has been tolerated well without any relevant adverse events. To prevent folic acid deficiency, the patient has received concomitant folic acid 10 mg orally, administered 24 h after each MTX dose. Routine CSF laboratory parameters, Q-IgM, and proportions of intrathecal IgM synthesis along tofersen treatment course are depicted in Fig. 1a, b.

### Neurofilament light chain levels and disease activity

Considering disease activity, NfL levels rapidly declined from 9911 to 5807 pg/mL following the tofersen loading phase, indicating the positive treatment effect of tofersen. Starting at T8, a backward trend was observed from 3141 pg/mL peaking to 4659 pg/mL by T13. Following administration of MP at T15 and T16, NfL levels gradually re-declined, up to that time reaching their lowest point during tofersen treatment at 2196 pg/mL by T19. However, by T20, levels rose again to 3210 pg/mL. Following MTX treatment, NfL levels decreased again and have remained consistently low, with 1481 pg/mL at T32 being the lowest concentration observed to date. At T1, the ALSFRS-R score was 37 out of 48. Following the loading phase, the score remained stable at 33 out of 48 through T7, suggesting no significant decline in motor function during the period of immune-mediated adverse events. Between T8 and T32, the mean ALSFRS-R score was 30 out of 48. NfL concentrations and disease activity along tofersen treatment course are illustrated in Fig. 1c.

## Discussion

This case highlights the clinical significance of autoinflammatory alterations in the context of tofersen treatment for *SOD1*-ALS, as such changes may even lead to therapy discontinuation [5, 6, 11, 13]. Whereas clinically apparent adverse events are rather rare, CSF abnormalities—predominantly pleocytosis and elevated CSF protein and intrathecal Ig synthesis—were commonly seen along the treatment course [10, 11]. However, little is known about the pathophysiological mechanisms, and the best approach to counteracting these events remains undefined. With tofersen recently being approved by the FDA and the EMA, there is an urgent need to understand the immunological impact of ASOs to better stratify the risk–benefit ratio, especially given the necessity of a lifelong treatment. This is particularly significant as the majority of patients with *SOD1*-ALS are likely to seek treatment with tofersen, and those receiving treatment reported a high level of satisfaction [3, 8]. In the presented case, a patient suffered from inflammatory reactions in CSF during tofersen treatment, most notably pleocytosis, intrathecal IgM synthesis, and impaired blood–brain-barrier function. Along the treatment course, she developed aseptic meningitis and neuralgia with radiological signs of polyradiculitis without any relevant decline in motor function. No spinal MRI scans with contrast were performed before tofersen treatment initiation. However, based on a recently published case report of a treatment-naïve patient with *SOD1*-ALS, contrast enhancement in the spinal nerve roots should be interpreted with caution [14]. Recurrent transient episodes of fever, rash, and fatigue following lumbar puncture may represent potential manifestations of a cytokine release syndrome (CRS) associated with ASO therapy. CRS has been documented with antibody-based therapies, non-protein cancer drugs, and stem cell transplantation [15]. However, it has not yet been reported in the context of ASO therapy. The initiation of an immunosuppressive therapy led to reduction of intrathecal inflammatory signs and alleviated clinical symptoms potentially linked to tofersen and its presumably autoinflammatory effect. Previous cases with immune-mediated adverse events were predominantly treated with corticosteroid regimens [5, 6, 11, 13]. However, their long-lasting effect remains speculative. In our case, corticosteroids provided only a temporary benefit, as pleocytosis and intrathecal immune activity relapsed after a short period. Subsequently, we initiated a concomitant oral MTX therapy leading to a sustained reduction of intrathecal pleocytosis, stabilization of intrathecal immune activity, and recovery of inflammation-related clinical symptoms. Of note, the induced autoinflammatory activity may negatively affect the disease-modifying efficacy of tofersen, as NfL levels seemed to be associated with pleocytosis and Ig synthesis. However, increasing NfL concentrations may be caused by the acute intrathecal inflammatory process itself, and decreasing NfL levels represent an effective anti-inflammatory treatment response. Following MTX treatment, NfL levels constantly decreased and remained on a stable low level as immune-mediated activity was attenuated. These alterations suggest that potential adverse autoinflammatory effects may influence the effectiveness of tofersen, and, possibly, disease progression.

## Conclusion

This clinical report presents a promising therapeutic approach for chronic immune-mediated adverse reactions induced by tofersen in patients with *SOD1*-ALS. While corticosteroids may provide short-term benefits, long-term concomitant treatment with MTX appears to have a sustained therapeutic effect. Although alterations in CSF may predominantly occur without clinical symptoms, the efficacy of tofersen may be altered by such autoimmune activities. Larger cohort studies are urgently needed to understand the immunological reactions in ASO treatments and to validate the long-term benefit of immunosuppressive treatment in patients suffering immune-mediated adverse events during tofersen treatment.

## Data Availability

The data that support the findings of this study are available from the corresponding author, upon reasonable request.
